# Essential Clinical Competencies for Novice Cancer‐Ward Nurses Before Independent Practice: A Modified Delphi Consensus Study

**DOI:** 10.1155/jonm/1177584

**Published:** 2026-11-06

**Authors:** Ching-Ching Lin, Chen-Yuan Lin, I.-Hui Chen, Tien-Li Liang, Yi-Chuan Cheng, Tzu-Pei Yeh

**Affiliations:** ^1^ Department of Nursing China Medical University Hospital Taichung Taiwan cmu.edu.tw; ^2^ Division of Hematology and Oncology Department of Internal Medicine China Medical University Hospital Taichung Taiwan cmu.edu.tw; ^3^ School of Pharmacy China Medical University Taichung Taiwan cmu.edu.tw; ^4^ School of Nursing Taipei Medical University Taipei Taiwan tmu.edu.tw; ^5^ Department of Nursing College of Nursing HungKuang University Taichung Taiwan hk.edu.tw; ^6^ Department of Nursing Asia University Taichung Taiwan asia.edu.tw; ^7^ School of Nursing China Medical University Taichung Taiwan cmu.edu.cn

**Keywords:** clinical competency, Delphi technique, novice nurse, oncology nursing, orientation

## Abstract

**Aim:**

To develop expert consensus on the clinical competencies that novice nurses must master before caring for cancer‐ward patients independently.

**Background:**

Oncology nurse managers commonly expect novice nurses to master cytotoxic handling, complex symptom management and emotionally demanding communication within weeks of orientation, yet evidence specifying what is essential before independent practice is limited.

**Methods:**

A three‐round modified Delphi study reported per CREDES guidance. Fifteen oncology experts (head nurses, advanced practice nurses, senior staff nurses and physicians) rated 92 items across four domains for importance and applicability over two questionnaire rounds; a face‐to‐face meeting between researcher and experts resolved remaining items. Consensus required mean ≥ 4.00, mode = 5, SD ≤ 1.00 and IQR < 1.00 on both scales. Data were collected February–June 2023.

**Results:**

Response rates reached 100% across all rounds. The final framework comprised 71 items in 33 subdomains (77.2% retention). Operational procedures retained 23/24 items (95.8%); communication retained only 1/7 (14.3%).

**Conclusion:**

The 71‐item framework defines what novice oncology nurses must master before independent practice and what should be developed progressively.

**Implications for Nursing Management:**

Nurse managers can use the framework to redesign orientation, allocate preceptor time and tie independent practice to demonstrated competence rather than elapsed weeks; the framework should be treated as a template requiring local validation rather than a directly transferable standard, given its derivation from a single‐region, three‐institution panel.

**Summary**
• What does this paper contribute to the wider global clinical community?
◦ A 71‐item, four‐domain competency framework that nurse managers can adopt to clarify which clinical skills novice oncology nurses must master before working independently and which can be acquired progressively during the first year of practice.
◦ Physicians, advanced practice nurses and senior staff nurses systematically differ in their expectations of novice nurses, underscoring the value of multidisciplinary consensus when designing orientation programs.
◦ The most pronounced reduction occurred in the communication domain (six of seven items removed); within this Taiwanese, task‐oriented orientation context, experts judged complex emotional and team‐representation skills to be unrealistic entry‐level expectations, though this finding may reflect local training culture rather than a universal developmental limit and should be interpreted accordingly in other settings.
◦ Nurse managers can use the framework, as a template requiring local adaptation and validation, to refine orientation schedules, set realistic competency benchmarks and support novice oncology nurse retention.

## 1. Introduction

The global nursing workforce continues to lose new graduates at alarming rates. In Taiwan, the licensure‐to‐practice ratio for registered nurses has dropped to approximately 63.6% and novice nurse turnover in medical centres exceeds 22.5%, reaching nearly 29% in regional hospitals [1]. Comparable attrition patterns disrupt care continuity, threaten patient safety and destabilize organizations across high‐income and low‐and‐middle‐income countries alike [2, 3]. Nurse managers everywhere are increasingly accountable for retaining a workforce that arrives at the bedside underprepared for the complexity of speciality practice. Among speciality settings, cancer wards stand out as particularly demanding: nurses there handle cytotoxic agents, manage chemotherapy‐related emergencies, perform complex tumour wound care, support patients through palliative trajectories and serve as the most consistent communicator within the interdisciplinary oncology team [4, 5].

Cancer‐ward novice nurses must absorb an extensive technical, cognitive and relational skill set within their first weeks on the unit. Preceptors and managers commonly expect newcomers to acquire every facet of oncology practice immediately, on the assumption that comprehensive competence equals safe care. The evidence, however, contradicts this assumption: novice nurses who feel underprepared report higher stress, more frequent errors and greater intent to leave [6]. Defining the subset of competencies that novice oncology nurses must master before independent practice—versus those they can develop over time—therefore, carries direct managerial value. Yet specific evidence on this question remains scarce.

Modified Delphi methodology offers a rigorous, efficient route to expert consensus when empirical trials are not feasible and when stakeholder perspectives diverge [7–9]. The approach suits the present problem because cancer‐ward practice is shaped by hospital‐specific protocols, professional guidelines and physician–nurse role boundaries that may not surface in any single clinical setting. By aggregating perspectives from oncology head nurses, advanced practice nurses, senior staff nurses and physicians across multiple institutions, a Delphi consensus exposes where disagreement exists and produces a defensible set of essential competencies.

This study used a three‐round modified Delphi design to identify the essential clinical competencies novice nurses must master before they care for cancer‐ward patients independently. We hypothesized that (a) expert consensus could be achieved on a structured competency framework spanning operational, cognitive, care‐delivery and communication domains and that (b) consensus would converge on operational safety while diverging on complex judgement and communication, reflecting realistic developmental sequencing for novice practice.

## 2. Background

### 2.1. Workforce Challenges and the Case for Competency Clarity

Novice nurse attrition reflects a mismatch between expected and actual readiness for practice. Drivers include unrealistic workloads, insufficient preceptor support, the steep learning curve of speciality knowledge and weak alignment between orientation curricula and bedside demand [2, 10]. For nurse managers, this is fundamentally a competency design problem: orientation programs that try to teach everything teach nothing well, while programs that defer competencies too long expose patients to risk. Clear competency definitions support targeted preceptor allocation, scheduling, performance evaluation and credentialing—all core managerial responsibilities.

### 2.2. Oncology‐Specific Competency Demands

Cancer‐ward nursing differs from general medical–surgical care in several quantifiable ways. Chemotherapy administration requires personal protective equipment, double‐check verification, infusion‐device management and competency in spill containment [11, 12]. Clinical‐trial drug administration adds documentation and monitoring requirements. Tumour wounds, complex central venous catheters (including implanted ports and peripherally inserted central catheters), neutropenic precautions and palliative cocare are routine. Communication demands are similarly distinctive: oncology nurses are positioned as continuity figures for patients and families, yet may be expected to manage emotionally charged conversations they have not been trained for [13, 14].

### 2.3. Modified Delphi Method for Nursing Competency Consensus

The Delphi method elicits structured expert opinion through iterative, anonymous rounds [15]. The modified Delphi method replaces the open‐ended first round with a literature‐derived structured questionnaire, accelerating consensus while retaining anonymity and statistical aggregation [8]. Researchers have used the approach successfully to define nursing competencies in palliative care, critical care and primary care contexts [16]; to our knowledge, however, no published modified Delphi study has defined competencies specifically required of novice nurses before independent oncology practice.

## 3. Methods

### 3.1. Design and Reporting

This study used a three‐round modified Delphi design to develop expert consensus on essential clinical competencies for novice cancer‐ward nurses prior to independent patient care. Data were collected between 10 February and 19 June 2023. The manuscript reports the study in accordance with the Conducting and Reporting Delphi Studies (CREDES) guidance [17]; the completed CREDES checklist appears in Appendix S1.

### 3.2. Setting

The study was conducted across three institutions in central Taiwan—one regional teaching hospital and two medical centres—all operating dedicated haemato‐oncology wards. The three institutions were selected to capture variation in case complexity, staffing model and orientation practice, factors that shape competency expectations for novice nurses. Including more than one institution mitigates the single‐site bias that limits the transferability of many Delphi competency frameworks.

### 3.3. Expert Panel Selection

Purposive sampling was guided by expertise criteria established for nursing Delphi research [8, 18]. Potential experts had to meet four criteria: (a) hold a current Taiwan nursing or medical licence; (b) work primarily in oncology practice for at least 3 years; (c) hold clinical teacher certification—the credential required in Taiwan to serve as a bedside preceptor for novice nurses—ensuring familiarity with novice education and competency assessment; and (d) be willing to participate in all three rounds. The team identified candidates through nursing‐director referral at each participating site and screened them for criterion fulfilment. Of 18 candidates approached, 15 met all four criteria and provided written informed consent.

The final panel size of 15 falls within the Delphi sample‐size range of 10–18 experts most commonly cited in the methodological literature [8, 19]; although this range was originally proposed for homogeneous panels, we applied it here only as a general size benchmark while deliberately constructing a heterogeneous panel spanning four oncology disciplines—head nurses (*n* = 2), advanced practice nurses (*n* = 4), senior staff nurses (*n* = 7) and physicians (*n* = 2)—because multidisciplinary panels surface role‐boundary assumptions that single‐discipline, homogeneous panels miss [7]. This is a deliberate departure from the homogeneous‐panel assumption underlying the original sample‐size guidance, made because role‐boundary variation was itself a phenomenon of interest. Demographic characteristics appear in Table 1.

**TABLE 1:** Demographic characteristics of the expert panel (*N* = 15).

Characteristic	*n* (%)	Mean	Range
Sex			
Female	14 (93.3)	—	—
Male	1 (6.7)	—	—
Age (years)	—	39.0	31–62
Years of oncology experience	—	13.0	3.0–30.0
Role			
Head nurse	2 (13.3)	—	—
Advanced practice nurse	4 (26.7)	—	—
Senior staff nurse	7 (46.7)	—	—
Physician (attending/chief resident)	2 (13.3)	—	—
Clinical teacher certification	15 (100.0)	—	—

*Note:* Experts were recruited from one regional teaching hospital and two medical centres in central Taiwan.

### 3.4. Instrument Development

The initial competency instrument was developed in two stages. First, two members of the research team conducted a structured literature review through PubMed, the Cochrane Library, the Airiti Library and the Taiwan electronic theses and dissertations system. Content‐focused searches combined ‘oncology nursing,’ ‘cancer ward,’ ‘novice nurse,’ ‘clinical competency’ and ‘orientation’ to retrieve competency evidence irrespective of methodology; a separate search adding ‘modified Delphi method’ was run only to identify prior Delphi‐based oncology competency frameworks for methodological benchmarking, not to constrain the competency‐content search. We nonetheless acknowledge that combining a methodology term with content terms in the same search strategy can orient retrieval toward Delphi‐derived competency lists and may have undersampled competency evidence reported through other methods (e.g., cross‐sectional surveys and qualitative studies); this is noted as a limitation in Section 5.5. Second, the team incorporated the third edition of the haemato‐oncology novice‐nurse work manual of one participating institution, supplementing it with national oncology nursing guidelines on cytotoxic drug handling, chemotherapy extravasation, central catheter care, mucositis, neutropenia and radiation skin care [11, 20].

The resulting questionnaire contained 92 competency items organized into 4 domains and 36 subdomains: (a) operational procedures (11 subdomains, 24 items, including admission, chemotherapy administration, bone marrow specimen handling, intrathecal injection assistance and chemotherapy spill handling); (b) work‐related cognitive ability (11 subdomains, 24 items, including laboratory report interpretation, oncological emergencies and neutropenia recognition); (c) nursing care including patient education (13 subdomains, 37 items, including radiation skin care, mucositis prevention, malignant wound dressing and port catheter care); and (d) communication (2 subdomains, 7 items, covering emotional assessment and team communication). Table S1 in the Supporting Information lists every item retained in the final framework.

Each item carried two five‐point Likert scales—importance (1 = *not at all important*, 5 = *extremely important*) and applicability to novice practice (1 = *not at all applicable*, 5 = e*xtremely applicable*)—plus a free‐text space for qualitative comments. Three nursing methodologists external to the research team reviewed the draft instrument for content validity; minor wording revisions followed their feedback. The content validity index for the revised instrument was 0.92 across reviewers, indicating acceptable face and content validity.

### 3.5. Delphi Procedure

The study followed a three‐round modified Delphi protocol (Figure 1). Round 1 (10–23 February 2023) used an online Google Forms questionnaire distributed by email. Round 2 (20 March – 7 April 2023) used a printed questionnaire delivered by registered mail; the format shifted because the volume of Round 1 qualitative feedback made online completion impractical for the marked‐up items; this cross‐round modality change is acknowledged as a limitation (Section 5.5). Both rounds included all 15 experts, with response rates of 100%. Round 3 (19 June 2023) consisted of a face‐to‐face consensus meeting attended by three discipline representatives—one head nurse, one advanced practice nurse and one chief resident physician—who volunteered to resolve items that had not achieved consensus after Round 2, selected for their supervisory or scope‐defining authority over these items. The seven senior staff nurses, who formed the largest professional group in Rounds 1‐2 (46.7%), were not represented at this final stage; we recognize this composition departs from Delphi anonymity and equal‐weighting principles and may have introduced hierarchical bias favouring supervisory and medical judgement, discussed further in Section 5.5. The discussion was audio‐recorded and transcribed to support audit‐trail transparency.

**FIGURE 1:**
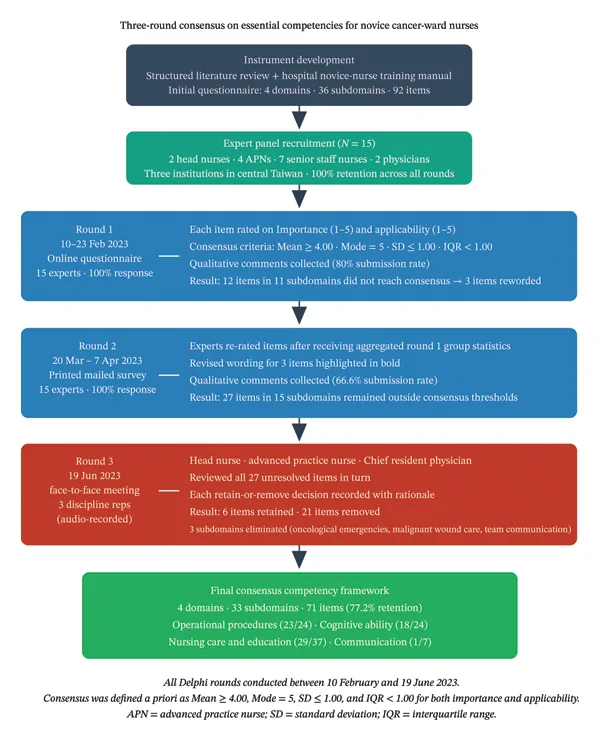
Modified Delphi study flow. Round 1 used an online questionnaire with 15 experts, Round 2 used a printed questionnaire with the same 15 experts after feedback and Round 3 used a face‐to‐face consensus meeting with three discipline representatives (one head nurse, one advanced practice nurse and one chief resident physician) to resolve items that had not reached consensus thresholds.

In Round 2, every expert received a summary of Round 1 group statistics together with revised item wording for items that had attracted substantive feedback. Revisions were highlighted in bold. Experts were instructed to reconsider their ratings in light of group results and qualitative comments, consistent with standard Delphi feedback procedures [7].

### 3.6. Consensus Criteria

Consensus was defined a priori using four criteria derived from established Delphi practice [8, 16]: (a) mean rating ≥ 4.00, (b) modal rating of 5, (c) standard deviation ≤ 1.00 and (d) interquartile range (IQR) < 1.00. Items that satisfied all four criteria for both importance and applicability were retained. Items failing one or more criteria were rerated in Round 2 and, if still not consensual, brought to the Round 3 face‐to‐face meeting for deliberation and a final retain‐or‐remove decision. The four‐criterion approach is more stringent than the single‐criterion (mean ≥ 4.00) approach used in many Delphi studies and provides additional protection against false‐positive consensus driven by central‐tendency rating patterns.

### 3.7. Data Analysis

Quantitative data were analysed with IBM SPSS Statistics Version 25.0 (IBM Corp., Armonk, NY, USA). Descriptive statistics summarised expert demographic characteristics. For each item, mean, mode, standard deviation and IQR were computed for both importance and applicability. The consensus criteria described above were applied to identify items requiring further discussion. Two researchers content‐analysed qualitative comments independently; discrepancies were resolved through discussion with a third researcher. Qualitative findings informed Round 2 item‐wording refinement and Round 3 deliberation.

### 3.8. Ethical Considerations

The study was approved by the Research Ethics Committee of China Medical University Hospital (approval number CMUH111‐REC3‐184; approval period 15 December 2022 to 15 December 2023). All experts received a written study information sheet describing purpose, procedures, voluntary participation, anonymity safeguards and data‐handling provisions. All 15 experts provided written informed consent before Round 1. Questionnaire identifiers were replaced with coded expert numbers to ensure inter‐round anonymity, and all electronic data were stored encrypted on a password‐protected institutional server accessible only to the research team.

## 4. Results

### 4.1. Expert Panel Characteristics

Fifteen experts participated in all three rounds (Table 1). Fourteen were female (93.3%). The mean age was 39.0 years (range 31–62) and the mean oncology experience was 13.0 years (range 3.0–30.0). The panel comprised two head nurses (13.3%), four advanced practice nurses (26.7%), seven senior staff nurses (46.7%) and two physicians (13.3%). All 15 experts held current clinical teacher certification, meaning the panel already comprised active oncology preceptors; academic educators from baccalaureate programmes were not separately recruited (see Section 5.5). Round 1 and Round 2 response rates were 100%, and all three Round 3 invitees attended the consensus meeting.

### 4.2. Round 1: Initial Expert Ratings

After Round 1, 12 of 92 items across 11 subdomains failed to meet consensus criteria for either importance or applicability (Figure 2). Areas of disagreement clustered in four locations: (a) detailed cognitive recognition tasks (interpreting low haemoglobin and platelet thresholds for medical reporting and urine pH monitoring for high‐dose methotrexate); (b) oncological emergency recognition (spinal cord compression, superior vena cava syndrome and tumour lysis syndrome); (c) catheter‐related judgement (identifying port‐catheter tip position on chest X‐ray) and (d) interpersonal and team communication. Twelve of the 15 experts (80%) submitted qualitative comments, from which three themes emerged: (a) some wording lacked clinical specificity and required revision; (b) several items appeared too advanced for novice practice and were thought to require time and case exposure and (c) interhospital protocol differences (for example, glove requirements during port dressing changes) generated dissonance about what was ‘standard.’ Three items were reworded for Round 2 (Table 2).

**FIGURE 2:**
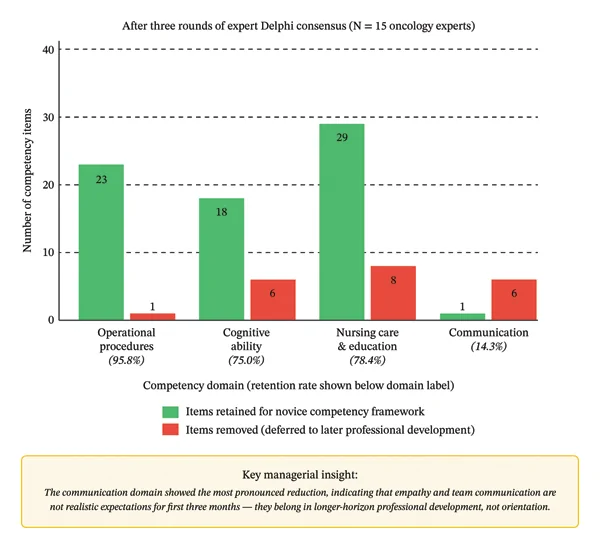
Number of competency items retained versus removed by domain after three rounds of expert Delphi consensus (*N* = 15 oncology experts). After Round 3, 71 of 92 initial items were retained across four domains; the communication domain showed the largest proportional reduction, indicating that complex relational competencies are not realistic for novice cancer‐ward nurses before independent practice.

**TABLE 2:** Items reworded between Round 1 and Round 2 based on expert qualitative feedback.

Domain/subdomain	Round 1 wording	Round 2 wording (revisions in italics)
Operational procedures/laboratory reports	Knows whether to notify the physician when haemoglobin or platelet counts are low.	Knows whether to notify the physician when any laboratory results are abnormal.
Cognitive ability/oncological emergencies—tumour lysis syndrome	Can explain that severe electrolyte abnormalities occur when potassium, phosphate and nucleic acids released from tumour cells exceed the body’s homoeostatic mechanisms.	Can explain that severe electrolyte abnormalities occur when potassium, phosphate and uric acid released from tumour cells exceed the body’s homoeostatic mechanisms.
Nursing care/intrathecal injection	Instructs the patient to remain supine for 6 hours after the procedure.	Instructs the patient to maintain a supine position initially after the procedure and to be closely monitored for any postprocedural discomfort.

*Note:* Revisions reflect expert feedback supported by current oncology guidelines.

### 4.3. Round 2: Rerating After Feedback

Round 2 narrowed but did not eliminate disagreement. After rerating, 27 items across 15 subdomains remained outside consensus thresholds. The communication domain showed the most persistent dissensus: six of its seven items had standard deviations above 1.10 for applicability and four of these had IQRs of 1.0 or greater. The cognitive ability domain accounted for the next largest cluster of nonconsensual items, particularly within oncological emergencies (three items), port and peripherally inserted central catheter management (one item) and chemotherapy extravasation (two items). Ten experts (66.6%) submitted qualitative comments at Round 2, predominantly reiterating that emotional judgement, oncological emergency recognition and tip‐position interpretation lay beyond realistic novice expectations and were better left to physicians or developed over time.

### 4.4. Round 3: Face‐to‐Face Between Researcher and Expert’s Consensus Meeting

The three Round 3 discipline representatives reviewed all 27 unresolved items in turn. The third round was conducted in face‐to‐face meeting between researcher and three experts independently. After two online Delphi rounds, most items reached consensus, but a small number remained contested, with systematic differences between disciplines. To resolve these, Round 3 was modified from an online questionnaire to a structured face‐to‐face consensus meeting focused only on the nonconsensus items. This allowed interdisciplinary clarification of meanings and real‐time negotiation of wording, an approach supported by previous modified Delphi studies that use in‐person meetings to finalize items when remote surveys are insufficient [9]. They agreed to retain 6 items and remove 21. Retained items addressed core safety procedures: bone marrow specimen preparation and transport, informed consent verification before intrathecal injection, aseptic assistance during intrathecal injection, skin decontamination after chemotherapy contact, urine pH monitoring rationale for high‐dose methotrexate and avoidance of routine blood sampling through port catheters. Removed items spanned three categories: (a) judgement‐heavy cognitive tasks better managed by physicians (oncological emergency interpretation and port tip‐position identification), (b) education topics with low patient adherence (postchemotherapy toilet flushing protocols) and (c) communication tasks that experts uniformly described as developmentally unrealistic for the first 3 months (empathic assessment and multidisciplinary case representation).

### 4.5. Final Competency Framework

The final framework retained the original four domains, eliminated three subdomains and produced 71 competency items across 33 subdomains (Table 3). Domain composition shifted markedly: operational procedures retained 23 of 24 items (95.8%); cognitive ability retained 18 of 24 (75.0%); nursing care with patient education retained 29 of 37 (78.4%) and communication retained only 1 of 7 (14.3%). The communication domain, therefore, showed the most substantial reduction, reflecting consistent expert judgement that empathy, team representation and complex emotional assessment require time and accumulated case experience and should be developed progressively rather than demanded before independent practice (Table 4). A subset of retained items (e.g., confirming informed consent and donning personal protective equipment) reflect institutional or legal requirements also seen outside oncology; these were retained not because consensus was needed to identify them in the abstract but because they are embedded in high‐consequence, oncology‐specific procedures (e.g., consent before intrathecal injection and PPE for cytotoxic handling) whose omission carries oncology‐specific safety risk. Table S1 in the Supporting Information presents the complete 71‐item framework with subdomain mapping, flags items of this type and supports nurse managers who wish to adopt the framework in their own units.

**TABLE 3:** Final consensus competency framework: items retained and removed across Delphi rounds.

Domain	Initial items	Items removed	Items retained	Retention (%)
1. Operational procedures	24	1	23	95.8
2. Work‐related cognitive ability	24	6	18	75.0
3. Nursing care (including patient education)	37	8	29	78.4
4. Communication	7	6	1	14.3
Total	92	21	71	77.2

*Note:* The full 71‐item retained framework with subdomain mapping is provided in Supporting Information Table S1.

**TABLE 4:** Round 3 expert decisions on items not reaching prior consensus (selected examples).

Domain/subdomain	Competency item	Decision	Rationale theme
Operational/bone marrow biopsy	Correctly prepares supplies and ensures correct bone marrow specimen submission.	Retain	Specimen integrity is nonrecoverable
Operational/chemotherapy spill	Manages skin contact with chemotherapy: removes contaminated clothing, washes with soap and copious water.	Retain	Nurse self‐protection
Cognitive/laboratory reports	Understands the rationale for monitoring urine pH in patients receiving high‐dose methotrexate.	Retain	Foundational drug‐safety knowledge
Cognitive/oncological emergencies	Recognises spinal cord compression from spinal metastasis requiring physician‐ordered corticosteroid therapy.	Remove	Physician scope; develops with experience
Cognitive/port catheter	Correctly identifies the port catheter tip position on chest X‐ray.	Remove	Physician scope
Nursing care/malignant wound	Selects dressing and antibiotics based on wound odour severity.	Remove	Physician orders; complexity exceeds novice scope
Communication/emotional assessment	Uses empathy to understand patient feelings.	Remove	Develops with time and case experience
Communication/team	Represents patient care issues during multidisciplinary team meetings.	Remove	Beyond first‐three‐month scope

*Note:* Decisions reflect unanimous consensus of the three Round 3 discipline representatives.

## 5. Discussion

This study produced a 71‐item, four‐domain competency framework that defines what novice oncology nurses should master before independent patient care—and, equally important, what they should not be expected to master in that period. To our knowledge, this is the first published modified Delphi study to address this question for the oncology context, and it surfaces three findings with direct managerial relevance for cancer‐ward leaders, though the specific item set and thresholds require local validation outside the study setting.

### 5.1. Operational Safety Dominates the Essential Set

Operational procedures emerged as the most stable domain, with 23 of 24 items retained. Experts converged tightly on chemotherapy handling, spill response, intrathecal injection assistance, transfusion protocols and bone marrow specimen integrity. This convergence aligns with national and international cytotoxic safety guidelines [11, 12] and reflects the consequence asymmetry of these tasks: a mishandled specimen, a contaminated intrathecal injection or a chemotherapy spill creates severe, often irreversible harm to either the patient or the nurse. Managers can act on this finding by front‐loading operational and safety competencies into the first weeks of oncology orientation and by structuring competency check‐offs around these high‐consequence procedures before allowing independent assignment.

### 5.2. Cognitive Judgement and Communication Require Developmental Sequencing

The most pronounced reductions occurred in cognitive judgement (six items removed) and communication (six items removed). Items removed from cognitive ability concerned oncological emergency recognition, port‐tip identification and selective extravasation judgement; items removed from communication concerned empathic engagement, psychosocial referral and case representation in multidisciplinary meetings. Experts consistently described these as competencies that develop through repeated case exposure, mentor dialogue and accumulated relational experience—not through orientation classroom hours. This finding is consistent with Benner’s novice‐to‐expert framework [21] and with subsequent oncology nursing competency work showing that interpretive and relational capacities lag behind procedural skill acquisition [22]. For managers, the implication is concrete: expecting empathic communication or oncological emergency triage from a three‐month novice creates a setup for failure that may accelerate rather than prevent turnover.

This pattern should, however, be read within its institutional and cultural context: orientation in the participating Taiwanese cancer wards has traditionally emphasized task‐based competency check‐off over structured relational‐skill development and several experts framed even basic empathic engagement, not only its advanced forms, as something that ‘develops with time.’ Studies show that empathy scores often increase across academic years, especially as students gain more clinical exposure and engage in structured reflection. Prior healthcare work and life experiences (e.g., caregiving and parenting) also correlate with higher baseline empathy in students [23, 24]. Therefore, although empathy is seen as an essential competency, it might not be completely established in novice nurse period.

### 5.3. Physician–Nurse Role Boundaries Shape Competency Expectations

The two physician experts and the four advanced practice nurses systematically moved several items out of the novice set on the grounds that responsibility for those tasks belongs to physicians or to more experienced nurses. Examples included tumour lysis syndrome recognition, spinal‐cord‐compression assessment and laboratory‐trigger interpretation for chemotherapy‐hold decisions. This pattern echoes earlier observations that multidisciplinary Delphi panels expose role‐boundary assumptions that single‐discipline panels miss [7]. Two implications follow for managers. First, novice nurse expectations should align with the actual scope of practice expected of them, not with an aspirational ceiling. Second, multidisciplinary input is essential when designing or reviewing speciality competency frameworks: a head nurse working alone is likely to assume nursing owns more of the work than physicians or advanced practice nurses agree it does.

### 5.4. Implications for Nursing Management

This study supports six concrete managerial actions, organised in ascending order of organisational reach. First, nurse managers can use the 71‐item framework (Table S1) as a structured orientation map: items map directly to learning modules, competency check‐offs and preceptor sign‐off criteria, replacing the open‐ended ‘learn everything’ model that drives orientation overload. Second, the framework supports more realistic scheduling decisions—managers can tie independent assignment to operational and core care competencies rather than to a comprehensive speciality profile, freeing preceptor capacity for the most critical safety items. Third, the framework gives managers an evidence‐informed basis to push back against organisational pressure for premature independent practice, supporting both patient safety and novice psychological safety. Fourth, the framework clarifies which competencies belong in continuing professional development beyond orientation, helping managers structure the first‐year learning trajectory as a developmental sequence rather than treating orientation as a single event. Fifth, by removing unrealistic communication and judgement expectations from the entry‐level set, the framework may reduce reality shock and contribute to retention; emerging evidence links structured, realistic onboarding to lower first‐year turnover and lower agency cost [25]. Sixth, the multidisciplinary derivation provides managers with a replicable template: managers seeking to adapt the framework in their own units can convene a similar mixed‐discipline panel to surface their own role‐boundary assumptions, rather than relying on a single‐discipline review. The underlying tension—comprehensive speciality knowledge versus realistic novice scope—is common across oncology settings, but the specific item set, thresholds and timing recommendations were derived from a central‐Taiwan, three‐institution panel; the framework should, therefore, be regarded as a template requiring local adaptation and revalidation rather than a directly transferable, ready‐to‐use standard.

### 5.5. Strengths and Limitations

Strengths include an a priori four‐criterion consensus definition more stringent than most prior Delphi studies, multidisciplinary representation across three institutions of differing complexity, 100% retention across all three rounds, systematic integration of qualitative feedback into instrument refinement and CREDES‐compliant reporting (Appendix S1).

Several limitations warrant acknowledgement. First, the panel of 15 experts, while within the Delphi sample‐size range of 10–18 commonly cited in the literature [8], was drawn from central Taiwan and may not capture practice variation across all Taiwanese regions or international healthcare systems; the multi‐institutional structure mitigates but does not eliminate this concern, and readers should consider local protocol differences when adapting the framework. Second, nursing educators from baccalaureate academic programmes were not included; although the panel already comprised certified unit‐level preceptors (Section 4.1), faculty perspectives could further enrich the linkage between prelicensure education and oncology orientation. This was a deliberate scope choice because the study focused on essential competencies for the immediate postorientation period rather than on curriculum design; future Delphi studies bridging prelicensure and postlicensure perspectives would meaningfully extend the findings. Third, the Round 3 face‐to‐face meeting departed from core Delphi principles of anonymity and equal‐weighted participation: only three discipline representatives (a head nurse, an advanced practice nurse and a physician) adjudicated the 27 unresolved items, and the seven senior staff nurses who formed the largest professional group in Rounds 1‐2 were not represented at this final stage. This composition may have introduced hierarchical bias favouring supervisory and medical judgement and could partly explain why several cognitive‐judgement and communication items were removed; future studies resolving residual Delphi disagreement should consider retaining proportional representation at the final consensus stage or explicitly analysing how panel composition relates to the direction of final decisions. Fourth, the data‐collection modality changed between Round 1 (online questionnaire) and Round 2 (paper‐based mailed survey); although intended to accommodate the volume of qualitative feedback, differing response modalities can themselves affect rating behaviour, and this cross‐round change is acknowledged as a methodological limitation rather than a neutral procedural adjustment. Fifth, the framework was derived from expert judgement rather than from prospective novice nurse outcomes; future research should test whether orientation programmes built around this framework demonstrably improve novice readiness, error rates and 1‐year retention. Sixth, although the panel included two physicians, a broader physician sample would further strengthen role‐boundary delineation. Seventh, importance and applicability ratings are subject to social desirability and to expert role identity; the multidisciplinary structure and anonymous Round 1‐2 rounds were designed to mitigate, but cannot eliminate, this concern. Eighth, combining the methodology term ‘modified Delphi method’ with content terms when identifying prior frameworks may have oriented retrieval toward Delphi‐derived competency lists, undersampling evidence from other methods (Section 3.4).

## 6. Conclusion

This modified Delphi study produced a 71‐item, four‐domain competency framework defining the essential clinical skills novice oncology nurses must master before independent patient care. Expert consensus converged on operational safety and core care skills as immediate priorities and diverged from the prevailing assumption that complex judgement and communication competencies must be achieved before independent practice. Nurse managers can use this framework to redesign orientation curricula, schedule competency check‐offs, allocate preceptor time and set realistic timelines—steps that may simultaneously protect patient safety, support novice psychological safety and improve early‐career retention in cancer wards. The framework’s strength lies in its multidisciplinary derivation; managers seeking to adapt it locally should consider replicating the multistakeholder approach to surface their own role‐boundary assumptions. The complete 71‐item framework appears in Supporting Information (Table S1) for direct adaptation.

## Funding

No funding was received for this manuscript.

## Conflicts of Interest

The authors declare no conflicts of interest.

## Supporting Information

Additional supporting information can be found online in the Supporting Information section.

## Supporting information


**Supporting Information** Table S1 presents a 71‐item competency framework for novice cancer‐ward nurses, developed through three Delphi rounds. It covers four domains: operational procedures, work‐related cognitive ability, nursing care and patient education and communication. It also provides suggested orientation timelines and a practical guide for nurse managers to plan training, assess competencies and determine readiness for independent practice. Appendix S1 is a supplementary CREDES reporting checklist for a modified Delphi study on essential clinical competencies for novice cancer‐ward nurses before independent practice. It lists 29 reporting criteria covering study design, expert selection, procedures, consensus, results, discussion and ethics and maps each criterion to its location in the manuscript. All criteria are marked as reported.

## Data Availability

The data that support the findings of this study are available from the corresponding authors upon reasonable request.
