# Supplementary material for: Essential Clinical Competencies for Novice Cancer‐Ward Nurses Before Independent Practice: A Modified Delphi Consensus Study
Source: J Nurs Manag. 2026 Nov 6;2026:1177584. doi: 10.1155/jonm/1177584 (PMC13743926; doi:10.1155/jonm/1177584)
Supplement: Supplementary file 1 [file JONM-2026-1177584-s001.docx]

*Supporting Information — Table S1*

**Essential clinical competencies for novice cancer-ward nurses before independent practice: A modified Delphi consensus study**

**Table S1. Full 71-item consensus competency framework**

This table presents the complete 71-item competency framework for novice cancer-ward nurses, organised by the four domains and 33 sub-domains that achieved expert consensus across three Delphi rounds. The "Suggested timing" column proposes an indicative competency-acquisition timeline (in weeks of orientation) for nurse managers to use when designing onboarding schedules. Timing recommendations are derived from expert qualitative feedback during the consensus process and represent typical expectations; managers should adjust to local context, novice prior experience and unit acuity.

Items are organised to support direct use as an orientation checklist: managers can assign each item to a preceptor sign-off cycle, link items to learning modules, and use the timing column to plan competency assessments before independent practice. Sub-domains within each domain follow the order used in the source instrument.

| **Domain 1 — Operational procedures (23 items across 10 sub-domains; retention 95.8%)** | | | |
| --- | --- | --- | --- |
| **#** | **Sub-domain** | **Essential competency item (novice nurse must demonstrate before independent practice)** | **Suggested timing** |
| 1 | Admission process | Understands the importance of measuring patient height and weight on admission for chemotherapy dose calculation. | Week 1 |
| 2 | Chemotherapy administration | Confirms that the chemotherapy consent form is signed before administration. | Week 1 |
| 3 |  | Correctly dons personal protective equipment for chemotherapy administration. | Week 1 |
| 4 |  | Confirms blood return and catheter security before administration. | Week 1–2 |
| 5 |  | Correctly calculates and adjusts IV pump infusion rates. | Week 1–2 |
| 6 | Laboratory reports | Confirms absolute neutrophil count (ANC) before chemotherapy administration. | Week 1–2 |
| 7 |  | Recognises abnormal laboratory results and notifies the physician appropriately. | Week 1–2 |
| 8 | Bone marrow biopsy | Confirms that the informed consent form is complete before bone marrow biopsy. | Week 2 |
| 9 |  | Correctly prepares supplies and ensures correct bone marrow specimen preparation and submission. | Week 2 |
| 10 | Intrathecal injection assistance | Confirms that the informed consent form is complete before intrathecal injection. | Week 2 |
| 11 |  | Uses aseptic technique to assist the physician with antineoplastic drug injection into the cerebrospinal space and correctly handles the CSF specimen. | Week 2–3 |
| 12 | Oral chemotherapy administration | Administers oral chemotherapy following the institutional standard procedure. | Week 2 |
| 13 |  | Manages chemotherapy-related waste correctly. | Week 1–2 |
| 14 | Opioid administration | Correctly draws up parenteral morphine. | Week 2 |
| 15 |  | Monitors for and manages opioid-induced constipation during opioid therapy. | Week 2 |
| 16 | Blood transfusion | Confirms transfusion consent before initiating blood product administration. | Week 1 |
| 17 |  | Monitors vital signs before transfusion, at 15 minutes after initiation, and after completion. | Week 1–2 |
| 18 |  | Recognises transfusion reaction signs, stops the transfusion immediately, notifies the physician, and follows blood bank protocols for haemolytic reactions. | Week 1–2 |
| 19 | Adult pain assessment | Reassesses pain 30 minutes after parenteral analgesia or 60 minutes after oral or non-pharmacological intervention. | Week 2–3 |
| 20 | Port-A and PICC catheter care | Performs daily CVC bundle documentation and assesses catheter status. | Week 2–3 |
| 21 | Chemotherapy spill response | Recognises the standard procedure for managing a chemotherapy spill incident. | Week 1 |
| 22 |  | Manages skin contact with chemotherapy: removes contaminated clothing immediately and washes with soap and copious water. | Week 1 |
| 23 |  | Manages eye contact with chemotherapy using the eye-wash station. | Week 1 |
| **Domain 2 — Work-related cognitive ability (18 items across 9 sub-domains; retention 75.0%)** | | | |
| **#** | **Sub-domain** | **Essential competency item (novice nurse must demonstrate before independent practice)** | **Suggested timing** |
| 24 | Laboratory reports | Understands the rationale for monitoring urine pH in patients receiving high-dose methotrexate. | Week 2–4 |
| 25 |  | Confirms ANC level before chemotherapy administration. | Week 1–2 |
| 26 | Opioid administration | Recognises the need for close vital-sign monitoring in patients receiving opioid analgesics. | Week 2 |
| 27 |  | Recognises precautions for fentanyl transdermal patch use. | Week 2–3 |
| 28 | Transfusion knowledge | Recognises that no medication may be added to blood products under any circumstance. | Week 1 |
| 29 |  | Recognises that blood products must be hung within 30 minutes of receipt and infused within the regulation-defined time limit. | Week 1–2 |
| 30 |  | Identifies the indications for leucocyte-depleted platelets (chronic transfusion patients, prior febrile non-haemolytic reactions, transplant or immunocompromised patients). | Week 3–4 |
| 31 | Myelosuppression | Recognises the definition of neutropenia, defined by absolute neutrophil count. | Week 2 |
| 32 |  | Interprets laboratory criteria for anaemia. | Week 2 |
| 33 |  | Interprets laboratory criteria for thrombocytopenia. | Week 2 |
| 34 | Oral mucositis | Uses the WHO five-grade oral mucositis assessment scale to recognise severity. | Week 3 |
| 35 | Malignant wound care knowledge | Assesses pain before dressing change and administers prescribed analgesia 30 minutes prior to dressing change. | Week 3 |
| 36 | Adult pain assessment knowledge | Selects appropriate pain assessment scales based on patient consciousness level. | Week 2 |
| 37 |  | Recognises the appropriate timing for pain assessment. | Week 2 |
| 38 | Port-A and PICC catheter knowledge | Recognises that a partially extruded catheter must not be reinserted into the vessel. | Week 3 |
| 39 | Chemotherapy extravasation prevention | Avoids placing peripheral IV catheters in the lower extremities because of poor peripheral circulation and drug accumulation risk. | Week 2 |
| 40 |  | Avoids multiple punctures of the same vessel to prevent extravasation. | Week 2 |
| 41 |  | Performs blood-return test before chemotherapy administration to confirm vascular access patency. | Week 2 |
| **Domain 3 — Nursing care including patient education (29 items across 13 sub-domains; retention 78.4%)** | | | |
| **#** | **Sub-domain** | **Essential competency item (novice nurse must demonstrate before independent practice)** | **Suggested timing** |
| 42 | Radiation skin care | Educates the patient on maintaining skin hygiene and selecting appropriate clothing during radiation therapy. | Week 3 |
| 43 |  | Educates the patient on managing pruritus and skin dryness. | Week 3 |
| 44 |  | Educates the patient on the importance of not removing or altering radiation skin markings. | Week 3 |
| 45 | Transfusion education | Educates the patient on transfusion-reaction symptoms and reporting precautions. | Week 2 |
| 46 | Post-chemotherapy GI side effects | Educates the patient on managing chemotherapy-related gastrointestinal side effects. | Week 3 |
| 47 | Oral mucositis care | Educates the patient to brush with a soft toothbrush at least twice daily. | Week 3 |
| 48 |  | Assesses oral pain and contacts the physician for topical analgesic prescription per protocol. | Week 3 |
| 49 | Bone marrow biopsy education | Educates the patient on post-biopsy wound care. | Week 2 |
| 50 | Intrathecal injection education | Instructs the patient to maintain a supine position after the procedure and to be closely monitored for any post-procedural discomfort. | Week 3 |
| 51 | Post-chemotherapy neutropenia care | Recognises that protective isolation precautions are required when ANC < 500/mm³. | Week 2–3 |
| 52 |  | Performs cluster care and adheres to the WHO five moments for hand hygiene. | Week 1–2 |
| 53 |  | Educates the patient on consuming thoroughly cooked food and avoiding raw food during the neutropenic period. | Week 2–3 |
| 54 | Adult pain non-pharmacological care | Provides non-pharmacological pain management measures. | Week 2–3 |
| 55 |  | Actively listens to and accepts the patient’s reports of physical pain. | Week 2 |
| 56 | Port-A catheter care | Performs aseptic Port-A catheter dressing and flushing. | Week 2–3 |
| 57 |  | Recognises flushing abnormalities (e.g., obstruction) and reports promptly. | Week 2–3 |
| 58 |  | Avoids routine blood sampling from the Port-A catheter to minimise infection risk. | Week 2–3 |
| 59 |  | Educates the patient on the importance of regular Port-A flushing to prevent obstruction. | Week 3 |
| 60 |  | Wears sterile gloves and mask when accessing the central catheter or changing the dressing. | Week 2 |
| 61 | PICC catheter care | Performs aseptic PICC catheter dressing. | Week 2–3 |
| 62 |  | Educates the patient on not measuring blood pressure on the PICC arm and on correct wound care. | Week 3 |
| 63 |  | Wears sterile gloves and mask when performing PICC care and dressing changes. | Week 2–3 |
| 64 |  | Educates the patient not to apply topical antibiotic ointment at the insertion site (fungal infection and resistance risk). | Week 3 |
| 65 | Chemotherapy patient education | Explains the chemotherapy regimen and schedule to the patient using approved educational materials. | Week 2 |
| 66 |  | Educates the patient to inspect oral mucosal integrity daily. | Week 2 |
| 67 | Chemotherapy extravasation education | Provides pre-treatment education on potential harm and discomfort symptoms of extravasation. | Week 2 |
| 68 |  | Instructs the patient to avoid leaving the ward during chemotherapy infusion to reduce extravasation risk. | Week 2 |
| 69 |  | Instructs the patient to report any abnormality or discomfort at the injection site immediately. | Week 2 |
| 70 | Post-chemotherapy GI care | Educates the patient on managing chemotherapy-induced diarrhoea and constipation. | Week 3 |
| **Domain 4 — Communication (1 item across 1 sub-domain; retention 14.3%)** | | | |
| **#** | **Sub-domain** | **Essential competency item (novice nurse must demonstrate before independent practice)** | **Suggested timing** |
| 71 | Initial emotional screening | Completes the emotional distress screening, suicide risk assessment, and psychiatric symptom screening tools within 24 hours of patient admission, per institutional protocol. | Week 1–2 |

***Note.*** *The framework retains the original four-domain structure. Three sub-domains were eliminated through expert consensus (oncological emergencies, malignant wound care, team communication); 21 items were removed and reassigned to longer-horizon professional development. See main manuscript Section 5.2 and Table 4 for rationale.*

***Note on item type.*** *A subset of retained items (e.g., #2, #8, #10, #16 — confirming informed consent; #3 — donning personal protective equipment) reflect institutional or legal requirements that also apply outside oncology nursing. These were retained not because expert consensus was needed to identify them as competencies in the abstract, but because they are embedded in high-consequence, oncology-specific procedures (e.g., consent before intrathecal chemotherapy injection, PPE for cytotoxic drug handling) whose omission carries oncology-specific safety risk; experts judged them essential prerequisite behaviours for independent oncology practice regardless of their generic legal origin. See main manuscript Section 4.5.*

**How to use this framework: a practical guide for nurse managers**

**Step 1 — Map to existing orientation modules.** Identify which of the 71 items are already covered in your current orientation programme. Items not covered should be added; items covered out of recommended sequence should be re-sequenced according to the suggested timing column.

**Step 2 — Assign preceptor sign-off owners.** For each item, designate a preceptor or clinical teacher responsible for direct observation and sign-off. Operational safety items (e.g., chemotherapy spill response, intrathecal injection assistance) should be signed off by certified clinical teachers; education items can be signed off by senior staff nurses.

**Step 3 — Schedule competency check-offs.** Use the suggested timing column to schedule formal competency assessments. We recommend at least three check-off points: Week 2 (operational safety bundle), Week 3 (core care and education bundle), and Week 4 (full framework review prior to independent assignment).

**Step 4 — Tie independent assignment to framework completion.** Defer independent patient assignment until the novice nurse has demonstrated all 71 items at the bedside, observed by a designated preceptor. This step is the strongest single managerial action this framework supports: it transforms independent practice from a calendar event into a competency event.

**Step 5 — Place removed items in continuing professional development.** The 21 items removed through expert consensus (oncological emergency recognition, complex extravasation judgement, empathic communication, multidisciplinary case representation) should appear in the unit’s first-year continuing professional development plan, not in the orientation programme. Most experts indicated these competencies develop over months to a year of accumulated case exposure.

**Step 6 — Re-validate locally if needed.** Managers in settings with substantially different protocols (for example, units that use different central catheter types or different chemotherapy bundles) should consider convening a brief local consensus panel — three to five experts representing nursing, medicine, and advanced practice — to validate the framework against local protocols before full adoption.

*Supporting Information — Appendix S1 (CREDES checklist)*

**Essential clinical competencies for novice cancer-ward nurses before independent practice: A modified Delphi consensus study**

*[Author names withheld for blinded peer review — see separate Title Page document.]*

**Appendix S1. CREDES reporting checklist**

The following table reports compliance of this manuscript with the Conducting and REporting DElphi Studies (CREDES) guidance for Delphi studies (Jünger, S., Payne, S. A., Brine, J., Radbruch, L., & Brearley, S. G. (2017). Guidance on Conducting and REporting DElphi Studies (CREDES) in palliative care: Recommendations based on a methodological systematic review. Palliative Medicine, 31(8), 684–706. https://doi.org/10.1177/0269216317690685).

Each CREDES criterion is mapped to the specific section, paragraph, table, or figure in the main manuscript or Supporting Information where the requirement is addressed. All 29 CREDES criteria are fully reported.

| **Section** | **Item #** | **CREDES criterion** | **Reported** | **Location in manuscript** |
| --- | --- | --- | --- | --- |
| **Rationale** | 1.1 | Description of the problem being addressed | **Yes** | Introduction §1; Background §2.1–2.2 |
|  | 1.2 | Justification of Delphi technique as the most appropriate method | **Yes** | Introduction §1 (paragraph 3); Background §2.3 |
| **Aims & objectives** | 2.1 | Clearly stated aim of the study | **Yes** | Introduction §1 (final paragraph); Abstract Aim |
|  | 2.2 | Specific, focused research question | **Yes** | Introduction §1; Abstract Aim |
| **Study design** | 3.1 | Outline of the study design | **Yes** | Methods §3.1 |
|  | 3.2 | Number and structure of rounds clearly defined | **Yes** | Methods §3.5; Figure 1 |
|  | 3.3 | Definition of consensus a priori | **Yes** | Methods §3.6 |
| **Expert panel** | 4.1 | Description of expert panel selection criteria | **Yes** | Methods §3.3 (criteria a–d) |
|  | 4.2 | Sampling strategy and recruitment process | **Yes** | Methods §3.3 (purposive sampling, nursing-director referral) |
|  | 4.3 | Number of experts and rationale for panel size | **Yes** | Methods §3.3 (15 experts; Keeney et al., 2011) |
|  | 4.4 | Characteristics of the experts (e.g., expertise, demographics) | **Yes** | Results §4.1; Table 1 |
| **Information given** | 5.1 | Information provided about the study purpose and process | **Yes** | Methods §3.8 (information sheet, consent procedure) |
|  | 5.2 | Information about anonymity, confidentiality, and feedback | **Yes** | Methods §3.8; §3.5 |
| **Procedure** | 6.1 | Description of the questionnaire and item development | **Yes** | Methods §3.4; Table S1 |
|  | 6.2 | Pilot testing and content validity assessment of the questionnaire | **Yes** | Methods §3.4 (3 external methodologists; CVI = 0.92) |
|  | 6.3 | Format and timing of rounds | **Yes** | Methods §3.5; Figure 1 |
|  | 6.4 | Feedback method between rounds | **Yes** | Methods §3.5 (group statistics, qualitative comments, revised wording) |
| **Consensus definition & attainment** | 7.1 | Definition of consensus | **Yes** | Methods §3.6 (four a priori criteria) |
|  | 7.2 | Method of consensus attainment and decision-making | **Yes** | Methods §3.6–3.7; Results §4.4 |
|  | 7.3 | Reporting of dropouts and response rates | **Yes** | Results §4.1 (100% retention); Methods §3.5 |
| **Results** | 8.1 | Presentation of results for each round | **Yes** | Results §4.2–4.4; Figure 2 |
|  | 8.2 | Presentation of changes between rounds | **Yes** | Results §4.3; Table 2; Figure 2 |
|  | 8.3 | Final consensus statements / outcomes | **Yes** | Results §4.5; Tables 3 and 4; Table S1 |
| **Discussion** | 9.1 | Discussion of findings in relation to existing literature | **Yes** | Discussion §5.1–5.3 |
|  | 9.2 | Strengths and limitations of the Delphi process | **Yes** | Discussion §5.5 |
|  | 9.3 | Generalisability and transferability of findings | **Yes** | Discussion §5.4 (final paragraph); §5.5 |
|  | 9.4 | Implications for practice or further research | **Yes** | Discussion §5.4; Conclusion §6; Table S1 practical guide |
| **Ethics** | 10.1 | Ethical approval reported | **Yes** | Methods §3.8 (IRB approval details on Title Page; withheld here for blinded review); Ethics Statement |
|  | 10.2 | Informed consent reported | **Yes** | Methods §3.8 (written informed consent before Round 1) |

*Note. All section, paragraph, table, and figure references map to the version of the manuscript submitted to the Journal of Nursing Management. CVI = content validity index; IRB = institutional review board.*
